# Supplementary material for: Osteo-pathological analysis provides evidence for a survived historical ship strike in a Southern Hemisphere fin whale (Balaenoptera physalus)
Source: PLoS One. 2023 Feb 22;18(2):e0281316. doi: 10.1371/journal.pone.0281316 (PMC9946264; doi:10.1371/journal.pone.0281316)
Supplement: Attachment — Submitted filename: Response to Reviewers.docx [file pone.0281316.s001.docx]

Response to Reviewer Comments

We would like to thank the reviewers for their thoughtful review and the constructive suggestions for minor edits in our manuscript. We have followed their advice and have revised the manuscript to implement the suggested changes. All changes are indicated by track changes in the revised manuscript. Line numbers in the response refer to the manuscript with track changes.

Reviewer #1: This is a compact and clear case study, providing results for basic research into cetacean pathology and its applied aspect in the welfare of whales. It also shows the increasing importance of "old fashoned" museum collections in research concerning threatened species which may be worth emphasizing in the text.

This is a useful suggestion, we have now added mentioning of the importance of museum collections in the discussion (lines 301-302)

The article is clearly written, supported by high quality illustrations obtained through sophisticated imaging methods.

We thank the reviewer for this very positive judgement.

Perhaps the description of directions could be clarified (actually simplified) in the foollowing figures:

Line 109: Figure 2: ... in lateral view from the external side
Line 113: Figure 3: ... in lateral view from the internal side.

I find the use of "lateral" confusing here as I suspect that the "external" side is lateral and the "internal" side is medial. I presume the original meaning was a reference to the lateral view of the skeleton in general. If I misunderstood something, a brief explanation would help other readers.

We agree. We have deleted 'in lateral view' in both figure captions. The captions now read: 'The [right / left] scapula of fin whale ZMH S 10261/10616 from the internal side'.

Line 140-143: Figures 8-9: ... left dorsolateral view

I think this is simply the lateral view as I do not see the vertebra being shown from "slightly above".

This is correct, we have removed 'dorso'

There seems to be superfluous wording here:

Line 147: Several lumbar vertebrae anterior (L5-L7) and posterior (L11-L15), [posterior and anterior of the...]

The text in square brackets could be deleted.

Yes, this was a mistake indeed. We have removed the words as suggested.

A reference could be added to a concrete parameters of HMS Ajax here:
Line 203: represents HMS Ajax, a ship of the time period estimated for the collision.

We have added the link to a webpage ('Naval History') presenting a detailed description of all ship specifics (line 218)

Although in the discussion proper references are made to a variety of accidents, it would be worth devoting a sentence in the text on how many similar traumatic cases are known. My impression is that this observation is quite unique, but it would be worth placing it in a slightly broader, concrete perspective.

Thank you for this idea. We have added a passage highlighting the rarity of a broken scapula observation in baleen whales at the beginning of the discussion, lines 176-183. In fact, there seems to be only one report of a broken scapula in a right whale

Reviewer #2: The submitted manuscript [PONE-D-22-27969] concerns the recognition of traumatic pathologies on the skeleton of a whale (Balaenoptera physalus) and the reconstruction of the traumatic event. The fractures were probably caused, according to the authors, by a collision with a ship in the 1940s. This whale was killed by a whaler in the South Atlantic in 1952 and its skeleton (ZMH S 10261/10616) belongs to the Hamburg Zoological Museum, which has been exhibiting it since 2015.

The authors used 2 types of 3D surface imaging: structured light scanning and photogrammetry. The interest of the 3D surface imaging used in this article lies mainly in the creation of a virtual model of the whale and the reconstruction of the traumatic event. Indeed, it does not help in retrospective diagnosis, as it does not offer more significant information than a set of good photographic images. A X-ray study of the damaged bones, for example, would have been interesting to visualize the internal bone structures, bringing additional information to the diagnosis of these consolidated fractures.

The adjustment of the different elements of the skeleton to propose a general 3D reconstruction of the whale, carried out by the authors according to their experience is interesting and useful to better understand both the location of the lesions and the history of the trauma. It seems to me to be well done, even if I am not a specialist in the anatomy of the whale skeleton.

We agree that an x-ray study of the bones would have been an interesting addition for an investigation of the internal bone structure. Unfortunately, we have no means of conducting such an analysis at our institute, particularly regarding the size of the bones. We are glad that the reviewer agrees that our approach using 3-D models was particularly useful for the assembly of the skeleton and reconstruction of the ship strike event.

My main comments are about some of the skeletal lesions and the interpretations:
- although there are no supporting radiographs, I agree with the authors on the diagnosis of healed fractures of the right scapula, the right ribs of ranks 1 and 2, and the lumbar vertebrae of ranks 8 to 10. The signs visible on the external surface of the 3D reconstructions of these bones are quite characteristic of traumatic conditions and do not require a differential diagnosis.

- the authors observed deformations of the spinous processes of the lumbar vertebrae located on either side of the fractured vertebrae, i.e. lumbar vertebrae of ranks 5 to 7 and 8 to 10. These deformations are interpreted by the authors as a condition due to posture. I would like the authors to better argue this interpretation : would they not be consolidated fractures of the spinous processes ? Indeed in figure 11, the orientation of the spinous process is very clear but could as well result from direct impact with the bow of the ship and not secondary to the animal’s posture due to pain.

We think the reviewer is referring to lumbar vertebrae 5-7 and 11-15, as theses are the ones deformed. We agree that our interpretation had been too brief and have now added additional argumentation. Firstly, we have added more detail to the description of the deformed vertebrae (line 147-149) and a new figure (figure 12) showing all deformations in detail. Secondly, we have added a whole paragraph of more detailed argumentation to the discussion (lines 266-273).

- similarly, the authors interpret the origin of the degenerative joint disease observed in the upper thoracic vertebrae (T2 to T6) as postural in origin. It would be interesting to illustrate these osteoarthritic vertebrae on the figures of the reconstructions of the animal's bone lesions (figures 1, 10, 13 and 14).

We have colorized the respective vertebrae in figure 10.

This would make it possible to discuss an alternative hypothesis: the degenerative phenomenon observed on the posterior vertebral joints at the thoracic level could be due, not to a postural cause, but to a joint contusion linked to the same trauma responsible for the fractures of the scapula and the ribs. Indeed, any joint trauma can induce a « contusive chondropathy » responsible for post-traumatic degenerative joint disease.

We greatly thank the reviewer for this suggestion. The proposed interpretation of chondropathy as a consequence of a joint contusion caused directly by the impact does indeed seem much more likely than arthrosis having been caused by posture damage, as previously suggested. We thankfully accept the reviewers suggestion and have changed our interpretation in the manuscript accordingly (lines 210-213).

- the authors mention in lines 194-195, that in the absence of bone histology data, it is difficult to determine the type of impact that caused the scapula fracture. If the authors believe that bone histology would differentiate between a blunt and a penetrating agent, I would like them to support their assertion here.

This was a mistake, we meant to only refer to histology of soft tissue. We have removed reference to bone.

- the authors propose an alternative hypothesis to the presence of two fracture sites on the whale at the scapula and the lumbar vertebrae (lines 224 to 227): indirect trauma by excessive flexion of the spine. I am not convinced by this hypothesis. The interpretation of a first impact on the whale's scapula with the ship and then a second impact when the whale swung its tail to dive seems more plausible.

We agree, that two consecutive impacts may be the more likely cause of events and we present this hypothesis as the first and most plausible one. However, we would still like to offer the alternative hypothesis, because there are interesting arguments supporting stress related fractures in cetacean vertebrae as a result of excessive bending and strains of the locomotor muscles (see Cooke et al. and Simões-Lopes et al. 2008).
