# Supplementary material for: Osteo-pathological analysis provides evidence for a survived historical ship strike in a Southern Hemisphere fin whale (Balaenoptera physalus)
Source: PLoS One. 2023 Feb 22;18(2):e0281316. doi: 10.1371/journal.pone.0281316 (PMC9946264; doi:10.1371/journal.pone.0281316)
Supplement: Attachment — Submitted filename: Response to Reviewers.docx [file pone.0281316.s002.docx]

Response to Academic Editor Comments

We would like to thank you for your review and the constructive suggestions for minor edits in our manuscript. We have revised the manuscript to implement the suggested changes. All changes are indicated by track changes in the revised manuscript. Line numbers in the response refer to the manuscript with track changes. We adjusted the reference list using the style guide PLOS in Endnote. We removed all figures from the manuscript and kept the captions only, presenting them following the respective paragraph.

1) There are inconsistencies in the writing of some terms: e.g., life history or life-history; 1950ies/1940ies or 1950s/1940s; post traumatic or post-traumatic; ship-strike or ship strike; 3D models, 3D-surface or 3-D model - please, revise the text and eliminate these inconsistencies;

Thank you for pointing out the inconsistencies in our manuscript. We have adjusted the following inconsistencies: We have changed life-history (lines 19 and 323) to life history; 1950ies and 1940ies to 1950s and 1940s (lines 20 and 29); post-traumatic to post traumatic (line 234) and ship-strike to ship strike (lines 34 and 302). We have also changed 3-D to 3D (lines 90 and 95) but we kept the following terms (3D model, 3D surface model and 3D skeletal model) as 3D surface model or 3D skeletal model is a specification of 3D model.

2) There are some typos or grammatical issues: e.g., line 76 – „or even not not suitable”; line 92 – „digital digiti”; line 166 – instead of its, their or the should be used; line 242 – „locomotors muscles” should be changed to locomotor muscles;

We have removed redundant words and corrected the mentioned typos and grammatical issues.

lines 246–247 – „on the vertebra’s spinal processus” should be rephrased; lines 269–270 – „as the ships size, speed and quantity” should be rephrased (e.g., as the size, speed and quantity of ships) - please, eliminate the aforementioned issues; and

We rephrased both lines as suggested. They now read: ‘on the spinal processus of the lumbar vertebrae L8-10’ (lines 273-274) and ‘as the size, speed and quantity of ships’ (line 299).

3) Figures and their captions: in the caption of figure 2, it seems that a space is missing between the specimen number and from; the caption of figures 6 and 7 should be rephrased to unify and simplify the captions (“The first right rib of fin whale ZMH S 10261/ 10616 in caudal view”; and “The second right rib of fin whale ZMH S 10261/ 10616 in caudal view.”); in the caption of figures 15, 17, and 18, “view” should not be capitalised; in the caption of figure 2, “of” is in a different font type than the rest of the caption; in the caption of figure 5, maybe external should be used instead of lateral, as in figure 2; in the caption of figure 9, one of the reviewers already mentioned that “dorso” should be deleted; and in figure 10, the “green” seems to me more like blue, and maybe the scapula should not be highlighted in red, as in the legend, only the vertebrae are mentioned.

Thank you for this suggestion. We have rephrased the captions of figure 6 and 7. Furthermore, we corrected the mentioned issues in the captions of figures 2, 5, 9, 15, 17 and 18. In the caption of figure 10, we changed the color description form ‘green’ to ‘blue’ and added a more precise phrase to include all fractured bones that are marked in red. We would like to keep the color of the ribs and the scapula in figure 10, as this figure is supposed to be a duplicate of figure 1 with additional information.
